# Changes in Microbial Plankton Assemblages Induced by Mesoscale Oceanographic Features in the Northern Gulf of Mexico

**DOI:** 10.1371/journal.pone.0138230

**Published:** 2015-09-16

**Authors:** Alicia K. Williams, Allison S. McInnes, Jay R. Rooker, Antonietta Quigg

**Affiliations:** 1 Department of Oceanography, Texas A&M University, College Station, Texas, United States of America; 2 Climate Change Cluster, University of Technology, Sydney, Australia; 3 Department of Marine Biology, Texas A&M University at Galveston, Galveston, Texas, United States of America; 4 Department of Wildlife and Fisheries, Texas A&M University, College Station, Texas, United States of America; Instituto de Biologia, BRAZIL

## Abstract

Mesoscale circulation generated by the Loop Current in the Northern Gulf of Mexico (NGOM) delivers growth-limiting nutrients to the microbial plankton of the euphotic zone. Consequences of physicochemically driven community shifts on higher order consumers and subsequent impacts on the biological carbon pump remain poorly understood. This study evaluates microbial plankton <10 μm abundance and community structure across both cyclonic and anti-cyclonic circulation features in the NGOM using flow cytometry (SYBR Green I and autofluorescence parameters). Non-parametric multivariate hierarchical cluster analyses indicated that significant spatial variability in community structure exists such that stations that clustered together were defined as having a specific ‘microbial signature’ (i.e. statistically homogeneous community structure profiles based on relative abundance of microbial groups). Salinity and a combination of sea surface height anomaly and sea surface temperature were determined by distance based linear modeling to be abiotic predictor variables significantly correlated to changes in microbial signatures. Correlations between increased microbial abundance and availability of nitrogen suggest nitrogen-limitation of microbial plankton in this open ocean area. Regions of combined coastal water entrainment and mesoscale convergence corresponded to increased heterotrophic prokaryote abundance relative to autotrophic plankton. The results provide an initial assessment of how mesoscale circulation potentially influences microbial plankton abundance and community structure in the NGOM.

## Introduction

Microbial populations are ubiquitous and abundant in the sea [1,2]. These organisms are characterized across all three domains of life and their immense diversity is reflected in significant contributions to many different marine processes [2,3]. Microbes that constitute the smallest size fractions of plankton, the pico and nano-plankton (0.2–20 μm), includes heterotrophic prokaryotes and dominant open ocean autotrophic species (e.g. *Synechococcus sp*. and *Prochlorococcus sp*.), which are believed to contribute >50% of biologically available carbon to oligotrophic systems [4]. The conversion of inorganic carbon to biologically available organic carbon by autotrophic microbial plankton directly or indirectly fuels abundant heterotrophic organisms, contributing to multiple energy transfer pathways and food webs [2,5]. Microbial cycling of fixed organic carbon in the euphotic zone is a major component of the biological pump and can ultimately alter the long-term sequestration of carbon in deep-ocean or marine sediments [6]. Therefore, understanding complex microbial contributions to various marine processes is important and remains a salient directive of current oceanographic research [7–9].

Marine microbial growth, production and activity are regulated by a variety of abiotic and biotic factors [9]. Both autotrophic and heterotrophic marine microbial plankton have multiple cellular requirements for inorganic nutrients [8] and therefore limitations by single or multiple nutrients can contribute to the overall microbial community dynamics [10,11]. Although not exclusive, typical limiting nutrients for plankton in the ocean are nitrogen, phosphorus, iron and, for some species, silica [8]. Identification of potential limiting nutrient(s) is important for understanding spatio-temporal controls on microbial communities [12–14]. The dominance of microbial plankton in oligotrophic regions is attributed to nutrient limitation prohibiting the growth of larger plankton. Alternatively, these regions may be a recognized niche for autotrophic prokaryotic plankton because they cannot out-compete larger phytoplankton in higher nutrient environments [3,15]. Physical mechanisms, such as mesoscale circulation, have been proposed to supply limiting nutrients to the euphotic zone in oligotrophic waters, potentially initiating planktonic responses, such as increased productivity and changes in community structure [16,17].

Mesoscale (50–200 km in diameter) cyclonic, anti-cyclonic and mode-water circulation patterns have been observed throughout the global oceans [18] and can cause pycno-, thermo-, and nutri-clines to dome upward or downward, depending on the direction of circulation [18]. In the Northern hemisphere, cyclonic and mode-water circulation are predicted to result in the upwelling of deeper water, supplying nutrients to the euphotic zone [18]. Several research initiatives have been developed to evaluate mesoscale circulation impacts on biochemical processes [16]. Diatom blooms were observed in the deep chlorophyll maximum in the cyclone *Opal*, formed leeward of the Hawaiian Islands in February, 2005 [19] and in mode-water eddies in the North Atlantic Subtropical Gyre (NASG) [20]. However, analysis of several cyclonic eddies in the NASG have shown a dominance of autotrophic prokaryotes at the deep chlorophyll maximum [17,20,21]. Bibby *et al*. (2011) propose that this discrepancy in the dominant plankton is caused by a difference in the availability of nitrate (NO_3_^-^) and silicate (Si[OH]_4_) supplied to the euphotic zone by upwelling in the NASG. Small microbial plankton can dominate the plankton community in cyclonic features when silicate is depleted relative to nitrate because diatoms require a 1:1 ratio [17]. The tracer Si*, which is the relative abundance of silicate [Si(OH)_4_]–nitrate [NO_3_^-^], has been shown to accurately reflect the dominance of phytoplankton communities in different eddy types [17].

In the Gulf of Mexico, mesoscale circulation associated with the Loop Current forms as the Caribbean Current enters the Yucatan Channel [22–25]. Cyclonic and anti-cyclonic eddies are often shed from the Loop Current [25], and these features can persist for at least 1.3 to 9.6 months [24]. Due to the narrowing of the continental shelf in the northeastern regions of the NGOM, mesoscale circulation interacts frequently with coastal shelf waters, including entrainment that can transport shelf water up to 300 km seaward [26–28]. This is particularly important in the region of the Louisiana/Texas where the Mississippi River and Atchafalaya River freshen and increase nutrient concentrations in continental shelf waters [28,29]. Dorado et al. (2012) examined phytoplankton, zooplankton and the N_2_ fixing cyanobacterium *Trichodesmium spp*. isotopic ratios to evaluate the contribution of different nitrogen sources to primary productivity across a low salinity plume (<32) and the anti-cyclonic circulation NGOM feature. Their findings show that plumes from the Mississippi River system impact N_2_ fixation, which subsequently influences pelagic food webs in the NGOM [30]. It is expected that mesoscale circulation upwelling eddies and coastal water entrainments will increase nutrient availability and therefore promote primary production.

The study described herein uses flow cytometry derived quantification and characterization of microbial plankton to evaluate their distribution across mesoscale circulation features of the NGOM. We hypothesize that the variability in the community composition is linked to the dynamic physicochemical conditions in the NGOM. Flow cytometry methods allow physiological (trait) based grouping of microorganisms and have recently been combined with multivariate statistical approaches to provide significant and important insights on changes in relative abundance of microbes and their potential ecological functions [31–34]. Although this study is only an initial assessment of the potential relationships between mesoscale circulation and microbial plankton in the NGOM, the results agree with what would be expected based on findings in other systems [19,35]. Data presented here establish a baseline to understand and predict the role of NGOM circulation features regulating microbial plankton community structure.

## Materials and Methods

### Study Area

**Fig 1:**
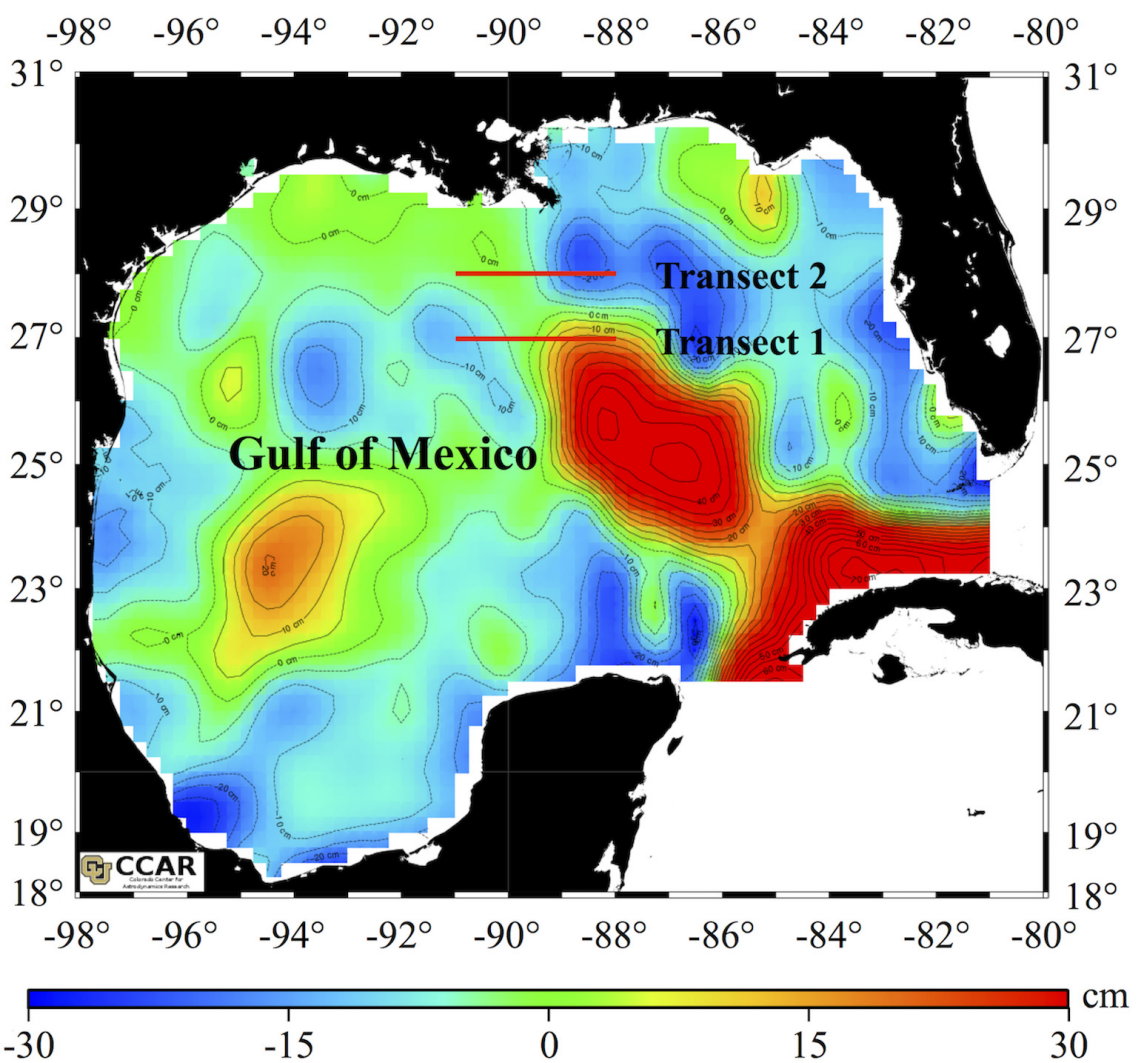
Map of the Gulf of Mexico showing the sea surface height anomaly (SSH) (cm). Cyclonic features are represented by cooler colors indicating negative SSH, while the anti-cyclonic loop current is represented by warmer colors indicating positive SSH. Black dashed lines delineate contours of similar SSH at intervals of 10 cm. Sampling transect 1 and 2 are highlighted in red lines.

A survey of microbial plankton (<10 μm) was conducted in the NGOM during a research cruise from 18 July to 22 July 2012 onboard the RV Blazing Seven. Thirteen stations were sampled along 27°N and 28°N (Transect 1 and 2 respectively; 26 total) running west to east from 88°- 91°W (Fig 1). No specific permissions were required for these locations/activities as we were collecting water samples on public lands. There was no animal research or other activities requiring any kinds of permits. Transects intersected cyclonic eddies and the anti-cyclonic Loop Current (Fig 1). The sea surface height anomaly (SSH) map for 20 July 2012 represents approximate conditions throughout the cruise. The map was generated from the Colorado Center for Astrodynamics Research (http://eddy.colorado.edu/ccar/data_viewer). We used satellite derived SSH data to predict the locations of mesoscale features with the understanding that there are inherent limitations to the resolution of circulation using this method. For example, satellite remote sensing of SSH does not provide information on vertical variability in eddy parameters through the water column, and cannot currently resolve rapid or small-scale (<100km) spatial changes [36]. Sea surface temperature (°C) (SST) was measured with a calibrated Conductivity Temperature Depth (CTD) sensor and salinity (unitless practical salinity scale) was determined with a calibrated Sonde 6920 Environmental Monitoring System (YSI Inc.).

### Sample Collection and Preservation

Seawater samples were collected from the surface (top 1 m) and depth (~30 m) into 20 L carboys and processed immediately. The ~30m depth target was selected to increase vertical resolution within dynamic eddy structures, but only this depth could be sampled due to timing and physical restrictions. All sampling equipment was cleaned with distilled water between stations and rinsed with sample water three times. Seawater was passed through a 20 μm mesh-size sieve into sterile 50 mL conical tubes containing 0.2 μm filtered paraformaldehyde and molecular biology grade glutaraldehyde at final concentrations of 1% and 0.01% respectively [37], and stored at -20°C until processing in a flow cytometer. The preservation method employed herein considered the premise that storage temperature (4°C or flash freezing to -80°C) has been found to have little effect on cell loss or histogram visualization [37]. Additionally, preservation biases are reduced when combining both paraformaldehyde and gluteraldehyde as fixatives [38].

### Nutrient Concentration Determination

Determination of dissolved nutrient concentrations followed a standard operating procedure established by The NELAC Institute, which does not enrich nutrients during filtration [39,40]. Seawater (50 mL) was filtered through a 0.7 μm glass fiber filter (Whatman, Kent, UK), and the filtrate was stored in sterile centrifuge tubes at -20°C until processing. The Texas A&M University Geochemical and Environmental Research Group determined concentrations of nitrate (NO_3_^-^), nitrite (NO_2_^-^), ammonium (NH_4_^+^), phosphate (P_i_−inorganic pool), silicate (SiO_2_), and urea from each water sample using an auto-analyzer (Astoria-Pacific, Clackamas OR) according to [41]. Resulting concentrations were quality checked against replicated standards and were significantly correlated (r ≥ 0.99). The tracer Si* was calculated by subtracting nitrate [NO_3_^-^] from silicate. The ratio of dissolved inorganic nitrogen (DIN) to phosphate (PO_4_-P) was calculated after summing the dissolved nitrogen inputs (DIN = NO_3_^-^ + NO_2_ +NH_4_^+^).

### Flow Cytometry

Heterotrophic and autotrophic microbial plankton groups were resolved using SYBR Green I staining procedures modified from [42] on a Gallios^TM^ 3-laser flow cytometer (Beckman Coulter, Brea, CA). The operational definition of microbial plankton cells used herein is based on a pre-filtration targeting cells <20 um in size and the frame of reference and volume used during flow cytometric processing. This method can capture individuals within both the pico- and nano-plankton size fractions (0.2–2 μm and 2–20 μm respectively) but most of the particles counted were smaller than internal standard beads used (<10 μm), indicating that the majority of these are pico-plankton (0.2–2 μm) cells (S1 Fig). Further, because in situ nano-plankton (2–20 μm) abundance is expected to be <1000 cells mL^-1^ [43] the volume of sample processed herein would not capture enough nano-plankton cells to significantly alter the total and relative abundances described. Aliquots of preserved sample were stained with 1/1000 diluted 10000X concentrated SYBR Green I (Invitrogen, Carlsbad, CA). SYBR Green staining and persistent fluorescence was enhanced by the addition of potassium citrate (30 mmol L^-1^ final concentration) to each sample [42]. Samples were incubated in the dark at ~60°C for 15 min. based on preliminary experiments which indicated increased binding efficiency of SYBR Green I at that temperature for the Gallios cytometer (data not shown). This incubation procedure did not cause variation in naturally occurring pigments prohibitive to group isolation. Internal size (10 μm) and enumeration (973 beads μL^-1^) standard flow count fluorophores were added to each sample tube post incubation (Beckman Coulter, Brea, CA.).

Particles were isolated within IsoFlow sheath fluid (Beckman Coulter, Brea, CA) and were exposed to 488 nm and 638 nm excitation by lasers and fluorescence was evaluated. Chlorophyll *a* emission was collected through a 695 nm band-pass filter ± 15 nm targeting its emission maximum of 667 nm. SYBR Green I emission was collected through a 525 nm band-pass filter ± 15 nm targeting its emission maximum of 522 nm. Phycoerythrin emission was collected through a 575 band-pass filter ± 15 nm targeting its emission maximum of 576 nm. Phycocyanin emission was collected through a 660 nm band- pass filter ± 15 nm targeting its emission maximum of 642 nm. Samples were analyzed for 5 min. at a flow rate of 4–8 μL min^-1^ discriminating on SYBR Green I fluorescence. Data analysis was conducted using Kaluza Cytometry Analysis software (Version 1.2 Beckman-Coulter, Brea, CA).

Autotrophic and heterotrophic cells were discriminated using Boolean gating on a combination of bivariate scatter plots (cytograms) or histograms with parameters including SYBR Green I, orange, and red fluorescence. Individual cells were grouped by the similarity in their physiological characteristics (S1 Fig) on the basis of previously reported observations of cultured and environmental samples [38,42,44–47]. Because the fluorescence of a group can vary through time and space [43], thresholds to standardize group identification among samples were identified based on the mean fluorescence of all the individuals within a group.

Heterotrophs found in the study area were separated into high nucleic acid containing bacteria (HNA) >2 (relative fluorescence units; RFU) and low nucleic acid containing bacteria (LNA) <2 RFU resolved with SYBR fluorescence (S2 Fig) and based on earlier descriptions [44,48]. We used nomenclature of A1-A5 for the 5 physiologically unique autotrophic groups present in our samples (S3 Fig). Group A1 maintained mean values of <10 RFU, <6 RFU, group A2 maintained mean values of >10 RFU, <6 RFU, group A3 maintained mean values of <10 RFU, >6 RFU, group A4 maintained mean values of >10 RFU, >6 RFU and group A5 maintained mean values of >10 RFU, >10 RFU for phycocyanin and phycoerythrin respectively (S3 Fig). Based on earlier studies [42,44,47], we can say the microbial plankton in this study are most likely *Prochlorococcus* (A1), *Synechococcus* groups lacking or containing different concentrations of phycourobilins, and pico-eukaryotic algae (A2-A5). However, taxonomic verification with molecular methods was not possible for the current study. Hence, we do not provide specific taxonomic identifiers as suggested in the recent reviews by [31,49] which detail both cautions and caveats of flow cytometry methods.

To quantify abundance (cells mL^-1^), the volume of each sample measured during flow cytometry was calculated by dividing the number of beads counted by the number of internal beads (uL^-1^) in the sample, and sample particle counts were divided by the calculated volume. To account for the addition of potassium citrate, SYBR Green and fixatives a dilution factor of 1:1.65 was applied to all values. To subtract background and noise, an aliquot of each sample was filtered through a 0.2 μm sterile syringe filter (VWR, Radnor, PA) and processed immediately after each sample.

### Statistical Analyses

Statistical analyses were performed using PRIMER V6.1.15 and PERMANOVA V1.0.5 [50,51]. All data were evaluated by draftsman plots in order to select appropriate transformation procedures and eliminate collinear variables. Non-collinear variables included in the analysis had correlations |r| ≤ 0.90, a more stringent threshold than |r| ≤ 0.95 as suggested by [51] in order to further reduce potential model bias while maintaining high resolution of variability within individual parameters. Transformation selections were further validated by comparison to both un-transformed data and data exposed to other transformation processes. *In situ* multivariate biotic data were transformed by log (1+y) in order to down-weigh the effects of a single group on the ordination and increase the contribution of rare groups [50]. All biological abundance data were analyzed using Bray-Curtis resemblance matrices. *In situ* multivariate abiotic environmental data for SST (°C), SSH (cm), salinity, nutrients (μg L^-1^) and ratios were square-root transformed in order to decrease skewness and increase linearity [50]. Transformed environmental data were then normalized to account for differences in units of measurement and analyzed using Euclidean distance resemblance matrices. Significant variability in combined abiotic parameters from each station were evaluated by Type III PERMANOVA main tests with unrestricted permutations of data. Principal coordinates ordinations were used to visualize similarities and dissimilarities in environmental conditions among stations. The largest Eigenvalue among factors identified the abiotic parameter most significantly correlated to each principal component [51].

Hierarchical cluster (CLUSTER) and similarity profile (SIMPROF, 97% similarity 9999 permutations) analyses were performed to cluster stations of similar microbial community abundance and composition. Significant differences in community structure were identified between the clusters by SIMPROF and verified by subsequent PERMANOVA pairwise tests (data not shown). Dendrograms were used to visualize statistical variability across stations. Non-parametric multi-dimensional scaling (nMDS) ordinations were used to visualize similarities and dissimilarities in microbial abundance and community composition among stations. The nMDS two-dimensional representation is considered acceptable for interpretations when the stress is less than 0.1 [50].

In order to quantify the relationship between measured environmental parameters and microbial community variability, predictor variables were identified using distance-based linear modeling (DISTLM). Models were generated using all possible combinations of predictor variable inputs with the “BEST” selection technique and both the Akaike information criterion corrected (AICc) and Bayesian information criterion (BIC). The top 10 models selected by each criterion test were plotted and overlapping models with the lowest AICc and BIC were considered [50,51]. The amount of variability in microbial community abundance and structure explained by environmental predictor variables identified by the model was quantified within DISTLM. Relationships between environmental predictor variables and communities were visualized using nMDS ordinations and Spearman derived correlated vectors.

## Results

### Environmental Conditions

Transect 1 at 27°N (stations 1–13) included areas of negative SSH consistent with cyclonic circulation, and positive SSH consistent with anti-cyclonic circulation associated with the Loop Current (Fig 1). Transect 2 at 28°N (stations 14–26) also included an area of pronounced negative SSH consistent with a cyclonic feature (Fig 1). At the surface, a distinct zone of relatively lower salinity oceanic water (average 37.1) was observed from stations 19–26 (S1 Table); at the remaining surface stations and all stations at depth (~30 m) salinity was on average >39 (S2 Table).

**Fig 2:**
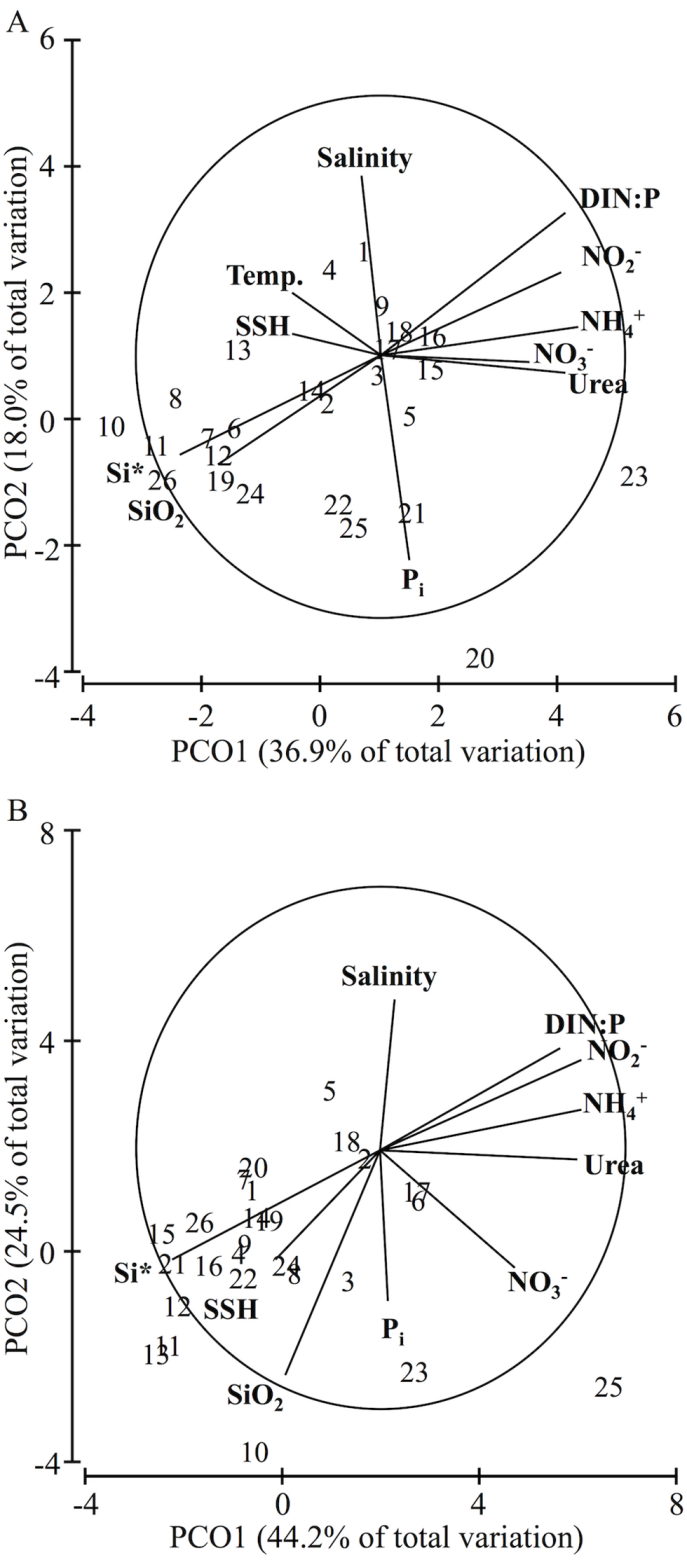
Spatial Variability of Environmental Conditions. Principal coordinate ordinations illustrating the variability of measured abiotic conditions across the 26 sampling stations. Spearman correlations for each abiotic parameter to the overall variability in environmental conditions are identified. Line length within the circle represents the relative strength of the correlation and direction corresponds to positive change. (A) variability (54.9%) in abiotic conditions for surface-water samples. (B) variability (68.7%) in abiotic conditions for deep-water samples.

All measured abiotic factors were included in principal coordinates and DISTLM analyses because none had correlations |r| ≥ 0.90 indicating lack of collinearity (S3 Table Table A and B). At depth, temperature was not recorded (S3 Table). Abiotic factors varied significantly at both surface (PERMANOVA main-test, n = 26, P(perm) = 0.0001, 9911 permutations) and depth (PERMANOVA main-test, n = 26, P(perm) = 0.0001, 9924 permutations). In surface water samples, 54.9% of the variability in abiotic conditions is explained by two principle coordinates axes (Fig 2A). Spearman correlation was strongest between PCO1 and Si* |r| = 0.82 and PCO2 and salinity |r| = 0.68. In deep-water samples, 68.7% of the variability in abiotic conditions is explained by two principle coordinates axes (Fig 2B). Spearman correlation was strongest between PCO1 and Si* |r| = 0.86 and PCO2 and salinity |r| = 0.87. SSH, Si* and Si were greater when DIN: P, NO_3_^-^, NO_2_^-^, NH_4_^+^, and Urea were lower in water samples measured at both depths (Fig 2). The concentrations of NO_3_^-^, NO_2_^-^, NH_4_^+^, and Urea were on average 1.2 times higher at stations with negative (-10 to -30 cm) SSH and, at the surface, on average 0.2°C lower SST (Fig 2). This is consistent with what is typically observed in cyclonic circulation features. Additionally, SST was on average 0.4°C higher at stations with positive (~10 to 30 cm) SSH (Fig 2). On average at surface and depth, Si was also 1.3 or 1.4 times higher with positive (~10 to 30 cm) SSH conditions (Fig 2).

### Distribution of Microbial Abundance

The study herein identified and quantified five autotrophic (A1-A5) and two heterotrophic (LNA and HNA) microbial groups (S1–S3 Figs). Total autotrophic cellular abundance (combined A1-A5 cells mL^-1^) varied between 7.1 x10^4^ and 2.4 x10^5^ cells mL^-1^ at the surface and 4.9 x10^4^ and 1.8 x10^5^ cells mL^-1^ at depth. Total heterotrophic cellular abundance (combined LNA and HNA cells mL^-1^) varied between 1.4 x10^5^ and 6.1 x10^5^ cells mL^-1^ at the surface and 1.1 x10^5^ and 6.8 x10^5^ cells mL^-1^ at depth. No significant difference (SIMPROF, n = 26, p ≥ 0.01) in total autotrophic or heterotrophic abundance was detected horizontally (between the stations) at either the surface or at depth.

Vertically, significantly higher concentrations of autotrophic cells (total) were present in surface waters when compared to depth (PERMANOVA, n = 51, p = 0.004) but there was no significant vertical variability in total heterotrophic abundance (PERMANOVA, n = 51, p = 0.521). Ranges in horizontal spatial distribution of individual groups are given for surface (S4 Table) and depth (S5 Table). Significant variability in the abundance of any individual group was not detected horizontally across surface (SIMPROF, n = 26, p ≥ 0.01) or deep stations (SIMPROF, n = 25, p ≥ 0.01).

### Distribution of the Microbial Community

**Fig 3:**
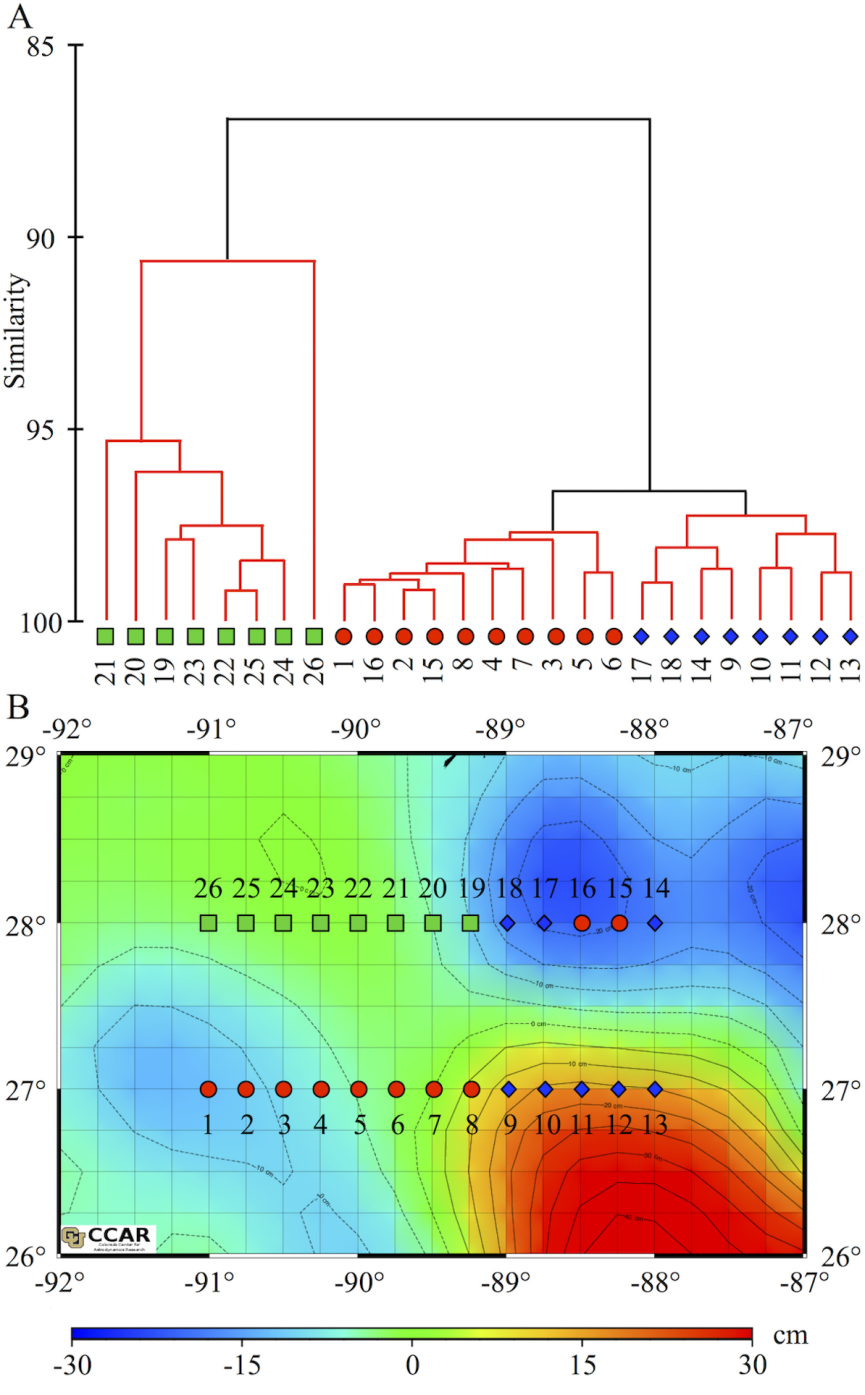
Spatial Variability in Microbial Community. (A) Group average cluster analysis based on the community structure. The black lines indicate that there is internal multivariate structure in the data at the 97% similarity threshold (p ≤ 0.01). Red lines indicate a non-significant test result; samples below this point are considered to have a homogenous community structure. Microbial signature one is represented by green squares, red circles represent microbial signature two and blue diamonds represents microbial signature three. (B) Map of sea surface height (cm) within the sampling region; contours of similar SSH delineated by black dashed lines. Symbols represent the 3 significant signatures of identified by CLUSTER and SIMPROF analyses. Cyclonic features are represented by cooler colors indicating negative SSH while the anti-cyclonic loop current is represented by warmer colors indicating positive SSH.

**Fig 4:**
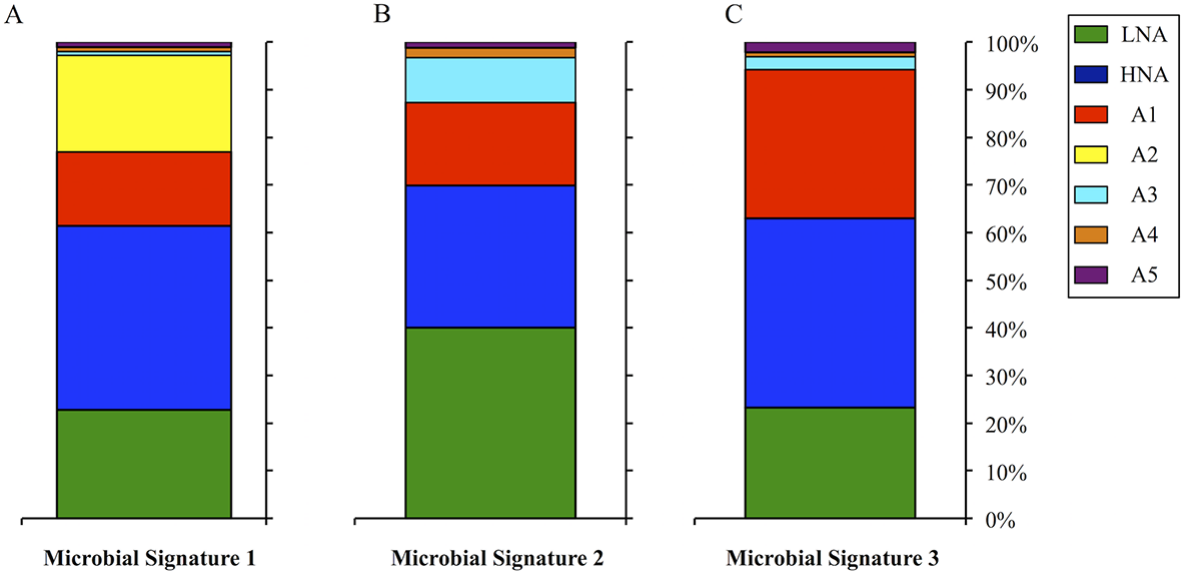
Microbial Community Structure. The relative proportion of autotrophic groups A1-A5 and heterotrophic groups LNA and HNA of representative samples for each of the three significantly different microbial signatures. (A) Microbial signature 1. (B) Microbial signature 2. (C) Microbial signature 3.

Community structure is herein defined as the proportion of each microbial plankton group A1-A5, HNA and LNA, compared to the total community at each station. Significant spatial variability (SIMPROF, n = 26, p<0.01) of community structure was detected across transects in surface waters (Fig 3A). Three microbial signatures (i.e. three unique community structure profiles, in each of which relative abundance patterns were statistically homogeneous) were identified (Fig 3A) and their locations related to mesoscale circulation patterns are plotted in Fig 3B. Representative samples showing the relative proportion of microbial groups associated with the three microbial signatures are plotted in Fig 4. No significant spatial variability (SIMPROF, n = 25, p>0.01) in the microbial plankton community structure was detected across transects at depth.

Microbial signature 1 was observed at stations 19–26 (Fig 3A). This signature was characterized by the presence of autotrophic group A2, which was not observed beyond this region (Fig 4A). Additionally, concentrations of autotrophic group A3 were more than four times less concentrated when compared to other stations (Fig 4A). Station 20, had the highest abundance of HNA of all evaluated stations (Fig 4A). Stations 19 and 20 had the highest concentrations of heterotrophic organisms observed in the study, two times greater than the average abundance observed at other stations. Microbial signature 2 was observed at stations 1–8 and 15–16 (Fig 3A). This signature was characterized by having two times higher concentrations of autotrophic groups A3, A4 and A5 and 1.2 times higher concentrations of HNA when compared to stations with microbial signature 3 (Fig 4B). Microbial signature 3 was observed at stations 9–13, 14, and 17–18 (Fig 3A) and was characterized by the lowest concentrations of autotrophic group A4, six times lower than other microbial signatures (Fig 4C).

### Statistical Correlation of Abiotic and Microbial Data

**Fig 5:**
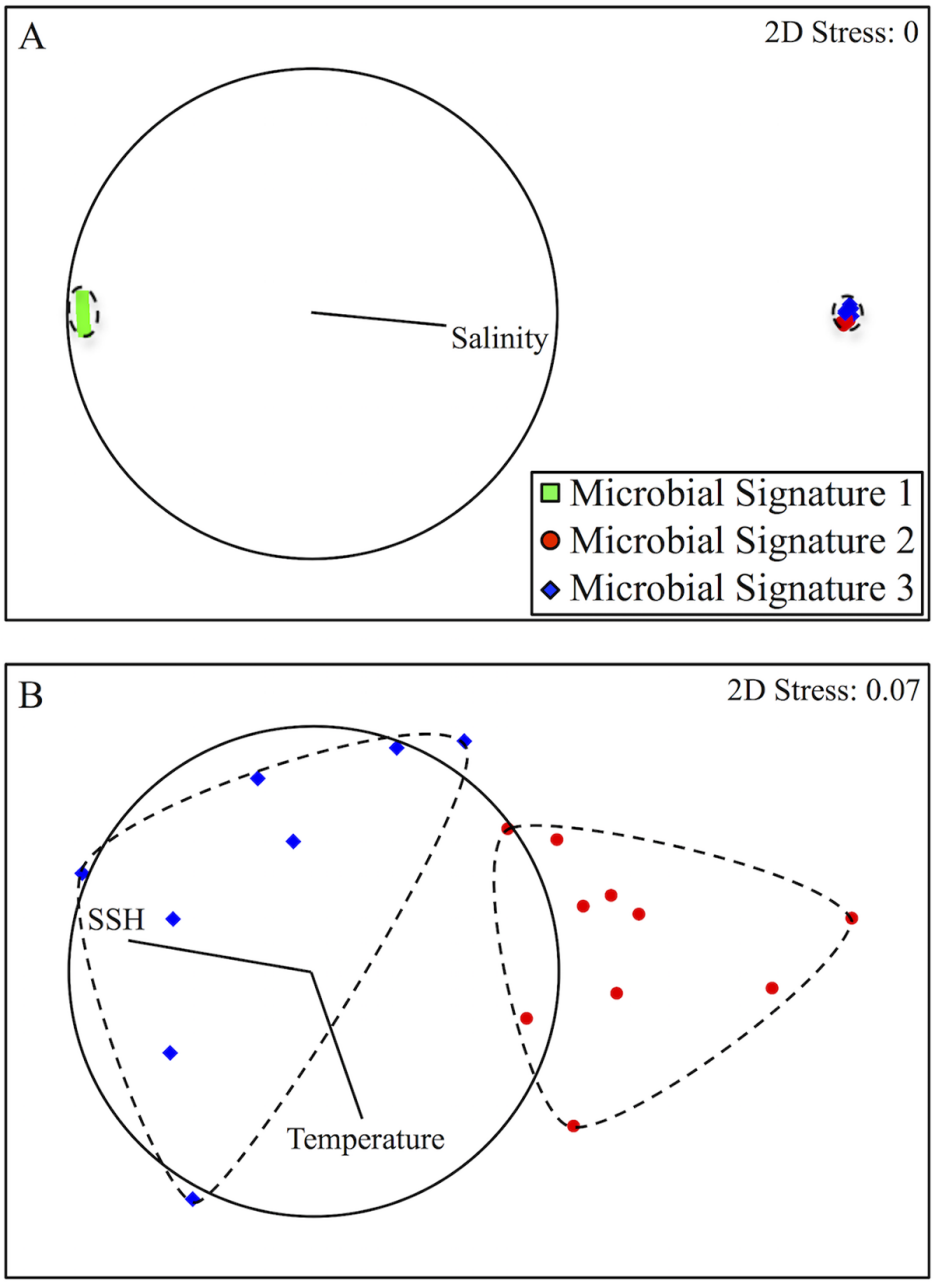
Significant Abiotic and Biotic Relationships. Non-parametric multi-dimensional scaling ordinations of variability in community structure between stations. The base variables identified by distance based linear models are Spearman correlated to the ordination. Vectors are plotted where line length within the circle represents the relative strength of the correlation and direction corresponds to positive change. (A) Separation of microbial signature 1 from 2 and 3. Dashed lines indicate 90% similarity of community structure. Forward R^2^, AIC_c_, and BIC values for salinity were 0.0001, 80.426 and 82.240 respectively. (B) Separation of microbial signature 2 from 3. Dashed lines indicate 97% similarity of community structure. Forward R^2^ values for SST and SSH parameters were 0.0002 and 0.1331 respectively. The AIC_c_, and BIC values for the combination of SST and SSH were 18.152 and 19.109 respectively.

Salinity was the most influential parameter in DISTLM explaining 54.7% of the variability in microbial plankton community structure (Fig 5A), and also the primary driver of the separation of microbial signature 1 from 2 and 3. Because stations with microbial signature 1 are located in a region of pronounced lower salinity, these stations were removed and DISTLM re-run in order to identify other abiotic parameters separating microbial signatures 2 and 3. A combination of SSH and SST was identified as the overall BEST solution in DISTLM explaining 56.0% of the variability in microbial plankton community structure at stations with signatures 2 and 3 (Fig 5B). Remaining abiotic variables tested individually explained <0.5% of the variation in microbial plankton community structure for both models.

## Discussion

Connectivity between physicochemical conditions of the study region and statistically unique microbial signatures (based on total and relative microbial group abundance measured using flow cytometry) was observed once multivariate statistical analyses were applied. While we do not know the specific constituents of these microbial communities (genomic analyses were not possible), based on previous flow cytometric and NGOM studies [44,48], the dominant microbial plankton present were likely *Prochlorococcus*, *Synechococcus* groups lacking or containing different concentrations of phycourobilins, pico-eukaryotic algae and two groups of heterotrophs (high and low nucleic acid). Importantly, the unique microbial signatures observed were associated with different water masses in the study site, each experiencing a unique set of coastal and/or mesoscale influences in the NGOM. Variations in nutrient availability associated with these mesoscale oceanographic features are not the only contributing factor to shifts in microbial community structure but were the focus of this study. Important examples (e.g. light availability, water stratification, competition and predation) have all been shown to influence microbial plankton [5,39,43,52,53] and should continue to be investigated by future studies in the NGOM.

### Microbial plankton in a region of freshwater entrainment

NGOM coastal ocean waters are subjected to large freshwater discharges from the Mississippi River and Atchafalaya River that can extend to our study region, beyond the continental shelf and into the loop current [29,54–56]. It has previously been observed that low-salinity coastal water entrainments affect phytoplankton community structure in the NGOM [30,57,58]. Additionally, the relative abundance of various nucleic acid containing heterotrophic microbial plankton groups was different between the Mississippi River plume and stations in the oligotrophic southeast GOM [48]. In this study, stations with microbial signature 1 are geographically adjacent to one another and are primarily located outside of the influence of mesoscale circulation. Physicochemically, stations with this signature are characterized by having the lowest observed salinity. Although there was a lack of significant statistical variability in microbial abundance or community structure at 30 m depth, DISTLM also predicted that salinity was the major driver of microbial variability below the surface (data not shown), further supporting entrainment of low-salinity coastal waters in the study region. Importantly, the lower salinity observed was still >32 indicating that water mass was oceanic, not neritic (<32) throughout the time of this study.

Several factors associated with coastal water entrainments in the NGOM may alter surface microbial community structure. The major shift in community structure observed was the presence of autotrophic group A2 exclusively at these stations (Fig 3A and 3B). This could be a result of specific halo-tolerance or because of a specific nutrient requirement that is met by the conditions present in the low salinity, high nutrient waters. Although variability in irradiance was not measured, light also could be a major factor influencing microbial community structure between a coastal water entrainment and the open ocean. Changes in light can result in shifts in the dominant photosynthesizing species or individual cells can adjust pigmentation to adapt to altering light conditions in the NGOM [39]. Additionally, water stratification derived from strong halocline gradients induced by coastal water entrainments could alter microbial plankton community structure [52]. Alternatively, competitive or predatory interactions could also be affecting abundance [9] and structure of microbial plankton. Importantly, the presence of this group indicates that entrainments can potentially supply resident coastal microbes to open ocean environments.

### Potential relationships between mesoscale circulation and microbial plankton

A significant relationship between microbial plankton community structure and a combination of SSH and SST was also detected (Fig 5B). These characteristics are frequently utilized to identify regions of mesoscale circulation [59–61]. SSH on its own, should not have a major effect on the microbial plankton community, as it is purely a physical measure. However, it has been documented that several physicochemical changes associated with SSH could impact microbial communities. For example, water temperature shifts associated with mesoscale circulation are extremely important to microbial organisms because physiologically, temperature can alter the kinetics of microbial metabolism and therefore the ability to compete and survive [9]. Models predict that in the open ocean temperature can limit productivity of heterotrophic microorganisms while nutrients are typically more limiting to autotrophic microorganisms [62]. The observed relationship between microbial plankton community structure and SST in this study indicates that thermal related limitations might exist for this size fraction of plankton. However, it is also possible that a different, unmeasured parameter is collinear with SSH and/or SST and is responsible for the observed relationship, such as light or vertical mixing depth.

#### Nutrient availability structuring microbial communities

Classically, it has been predicted that limiting nutrients will be made available to the euphotic zone by doming pycnoclines in cyclonic circulation supporting higher abundance and productivity of autotrophic and heterotrophic plankton [18,63,64]. At two stations, 13 and 16, corresponding mesocosm experiments were conducted and results verified that significant shifts in the microbial plankton community structure occurred within 24 hours of enrichment with nitrate supporting that nitrogen limitation of microbial plankton potentially exists in this region (data not shown, p≤0.01 at both stations, Monte Carlo PERMANOVA, partial type III pairwise test). However, in this study, DISTLM did not identify any of several growth limiting inorganic nutrients tested as having a significant relationship with microbial plankton community structure. There are several potential explanations.

First, it is possible that mesoscale circulation did not facilitate significant nutrient enrichment to surface waters targeted in this study. This is supported by a lack of significant collinearity between nutrients and SSH or SST. Previous research has observed that cyclonic eddies formed along the Leeuwin Current can be capped by warm Indian Ocean waters limiting upwelling from reaching the shallow euphotic zone [65]. A similar circumstance may potentially be occurring in the NGOM as less dense coastal waters could cap upwelling in cyclonic features. Additionally, the intensity of pycnocline doming in mesoscale circulation features varies throughout their life cycle [18] and has been observed to shift regularly in GOM eddies [66]. Therefore, because the relationship between eddies and the community structure of microbial plankton is not clear, future experimentation should be conducted to better capture integrated vertical and horizontal nutrient variability, to determine potential connectivity to microbial plankton at a higher resolution.

Second, it is important to note, that several abiotic and biotic factors can influence microbial abundance, community structure and functions [9], most notably grazing which was not examined in the current study. Therefore, the specific factors identified herein are likely not the only drivers of changes in the overall microbial abundance. It is possible that the size fractions of plankton targeted in this study are not being directly influenced by nutrient availability but are indirectly influenced by it. If larger phytoplankton are limited by nitrogen in this region, which becomes available in the euphotic zone where mesoscale circulation is occurring, they might increase in abundance and compete with microbial plankton [9,67].

**Fig 6:**
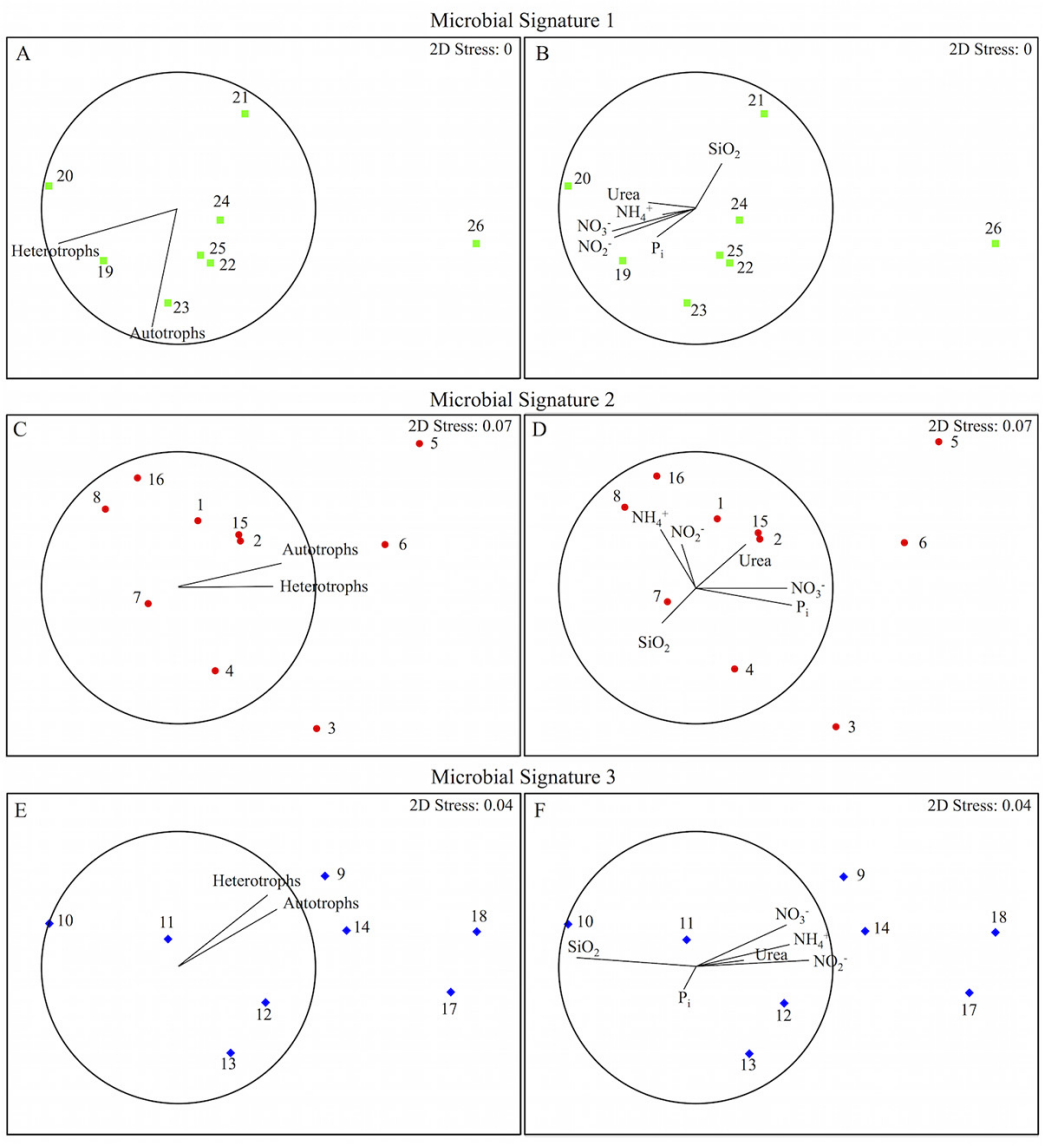
Ordinations of Nutrient/Microbial Abundance Relationships. Non-parametric multi-dimensional scaling ordination of variability in community structure between stations within each of the three significantly different microbial signatures. Spearman correlations between total autotrophic and heterotrophic microbial plankton abundance (A, C, E) and between measured nutrient concentrations (B, D, F) for those stations are both plotted as vectors, where line length within the circle represents the relative strength of the correlation and direction corresponds to positive change. (A, B) Variability of community structure at stations within microbial signature 1. (C, D) Variability of community structure at stations within microbial signature 2. (E, F) Variability of community structure at stations within microbial signature 3.

Third, it is possible that the variability in nutrient concentrations was too small to be considered significant by the DISTLM method when related to physiologically based groupings of microbial plankton, which is a potential limitation of flow cytometry derived community structure. To address these possibilities and increase the resolution of specific mesoscale impacts, nMDS ordinations of stations within each microbial signature group were correlated to nutrient availability and total microbial autotrophic and heterotrophic abundance rather than community structure using Spearman statistics (Fig 6). Important trends were observed based on Spearman vectors, suggesting that the strict DISTLM/community structure analysis of overall nutrient: microbial relationships may be limited in capturing all relevant connections and it highlights the importance of considering several statistical approaches to validate findings when conducting flow-cytometric analyses.

#### Nutrient availability influencing microbial abundance

Potential relationships between microbial abundance and nutrient availability, specifically DIN, were detected across microbial signatures (Fig 6). Stations 21, 4 and 7, and 10–13 had the lowest microbial plankton abundance among stations within their respective microbial signatures (Fig 6A, 6C and 6E). Lower concentrations of DIN and sometimes P_i_ were measured at these stations (Fig 6B, 6D and 6F). Within groups of stations with the same microbial signature, stations with limited DIN and P_i_ were typically located either in regions outside of mesoscale circulation or in regions of positive SSH, likely associated with anti-cyclonic features (Fig 3B). These findings support previous analyses suggesting that, in oligotrophic waters off of the Louisiana/Texas shelf, N and/or P_i_ are the main limiting nutrients to primary producers [29,39,58]. Interestingly, the proportion of LNA:HNA was the highest at stations 10–13 (microbial signature 3) within the study region. This potentially indicates that the heterotrophic bacterial community within the anti-cyclonic Loop Current is not only low in abundance but also less active [68]. Alternatively, it has been proposed that cells with small genomes are more efficient at nutrient uptake and use [69]. Therefore, the increased proportion of LNA could be an adaptation of the community to reduced availability of nutrients or a result of limited organic carbon produced by larger autotrophs in this low-nitrogen region.

A negative relationship was observed between Si and DIN (Fig 6B, 6D and 6F). This observation implies that Si could be a limiting factor at stations where N and P_i_ are available [70]. The lower concentration of Si corresponding to increased DIN supports the premise that silicate-consuming organisms may be present at these stations. Previous research has shown that mesoscale circulation can induce blooms of diatoms [67,71] and a succession from microbial plankton to larger diatoms in central, high nutrient regions of cyclonic circulation features [71]. However, in the NGOM, the abundance of microbial plankton increased in regions of N availability and Si limitation, indicating that they are not competing with larger organisms for limiting resources. One potential explanation is the recent finding that *Synechococcus* spp. contains high levels of silica under certain conditions [72]. Although the reasons and mechanisms for uptake are not yet described, it is possible that the observed decreased Si concentration is the result of picocyanobacterial uptake of Si when nitrogen is available. Increased microbial plankton abundance associated with decreased Si but increased NO_3_^-^ supports the theory proposed by [17] that, unless Si* is optimal for diatom growth, microbial plankton will dominate in response to mesoscale circulation rather than diatoms. Alternatively, it is possible that a less severe succession in the circulation features in the NGOM was driven by different responses of resident populations. Overall, these observations suggest that shifts in nutrient limitation across mesoscale features have important implications for the ecology of the plankton community.

#### Microbial abundance in mesoscale frontal convergence zones

Frontal convergence zones have previously been identified as ‘hot spots’ for microbial abundance and productivity, associated with the shallowing of nutriclines [63,71]. In this study, several examples of increased abundance were observed at stations where increased upwelling in frontal zones is expected. Each of these examples was correlated to higher concentrations of NO_3_^-^, NO_2_^-^ and NH_4_^+^, indicating that microbial plankton are nitrogen limited. An example is station 9, located along the western frontal zone of the anti-cyclonic Loop Current, which has the highest abundance of microbial plankton among stations with microbial signature 3 (Fig 6E). Previous studies have shown that upwelling occurs at the margin of anti-cyclonic features [21,60] and in anti-cyclonic eddies in the GOM [66]. Because concentrations of NO_3_^-^, NH_4_^+^ and NO_2_^-^ were higher at this station (Fig 6F), it is possible that the upwelling caused a shift in nutrient concentrations that consequently increased the abundance of microbial plankton at this location. In addition, stations 14, 17, and 18 were located in a northern cyclonic mescoscale feature where typical cyclonic upwelling supported the highest concentrations of inorganic nitrogen and the corresponding higher microbial plankton abundance (Fig 6F).

### Combined physicochemical influences on microbial plankton

A unique set of conditions was observed in the northern cyclonic feature (Fig 1) because parts of the frontal convergence zone of this feature were also located within a lower salinity region. It has been proposed that lower salinity coastal water entrained into mescoscale circulation could supply different plankton species to these features [73]. Two stations, 19 and 20, were located in the region where both mesoscale features and coastal influences were observed. These stations have the second and third highest total microbial plankton cellular abundance observed across the study region. Resident coastal microbial plankton entrained into mesoscale upwelling zones potentially respond more quickly to mesoscale nutrient pulses than organisms acclimated to oligotrophic conditions, resulting in the observed higher abundance. At these stations, 10% more heterotrophs were present than elsewhere in the study region. Additionally, these stations had the highest concentrations of HNA, which may have higher metabolic activity [9,68].

Competition for dissolved organic matter can occur between autotrophic and heterotrophic prokaryotes in the microbial plankton [9]. The observed heterotrophic increase indicates that this region of combined nutrient enrichment from mesoscale circulation and coastal waters could shift microbial processes to net heterotrophy.

### Potential implications of significant spatial variability in microbial plankton

The ecological implications of mesoscale driven shifts in microbial plankton abundance are particularly relevant to higher tropic consumers. Successful recruitment of fish larvae to adult populations is imperative in maintaining sustainable fisheries [74,75]. Several studies have proposed the match/mismatch hypothesis linking availability of plankton prey sources to eventual successful recruitment [74]. Frontal convergence zones along the northern margin of the anti-cyclonic Loop Current in the NGOM represent important early life habitat of several pelagic fish species including billfishes, tunas and swordfish [76,77], indicating that this region is an important spawning or nursery area [56]. The increased abundance of microbial plankton observed in this study at stations near to the frontal zone of the Loop Current suggest a mesoscale induced increase in microbial plankton potentially contributes to bottom-up processes along the margin of this feature. Additionally, spatial dynamics of the Loop Current vary temporally resulting in changes in the larval distribution [40], which can be linked to availability of prey. Therefore, future studies should continue to evaluate microbial plankton responses to circulation at a high resolution throughout eddy features, which is necessary to better understand trophic relationships and other potential impacts of changing microbial abundance in the Northern Gulf of Mexico.

## Supporting Information

S1 Fig Group Identification Procedure used to isolate individual microbial groups with KALUZA for a representative sample. The magnitude of fluorescence is plotted on a logarithmic scale and colored lines represent the percentage of total count: red is 80%, green is 60%, and blue is 40%. (A) All particles counted with cells distinguished from the internal standard of beads. (B) Cells counted are plotted using chlorophyll *a* and phycocyanin fluorescence. Heterotrophic cells (Gate 1) are distinguished from autotrophic cells (Gate 2). (C) Autotrophic cells are plotted using phycoerytrhin and phycocyanin fluorescence. Groups 1 and 2 are distinguished from each other based on relative phycocyanin fluorescence. (D-H) As an example, we show Group 2 cells plotted against all other measured fluorescence parameters (forward scatter, SYBR Green I, chlorophyll *a*, phycoerythrin and phycocyanin), to verify consistency in physiological characteristics among individuals.
(PDF)

S2 Fig Heterotrophic Group Isolation. Particle count (y-axis) across the spectrum of SYBR Green I fluorescence (x-axis, plotted in a logarithmic scale) classified as heterotrophic cells using flow cytometry. The delineation between lower-nucleic acid containing bacteria (blue) and relatively higher nucleic acid containing bacteria (green) is marked by a black dashed line.
(PDF)

S3 Fig Autotrophic Group Isolation. Autotrophic groups in representative samples for three significantly different microbial signatures. The magnitude of fluorescence is plotted on a logarithmic scale (x and y axes) and colored lines represent the percentage of total count. The mean relative phycocyanin fluorescence (x) and mean relative phycoerythrin fluorescence (y) for individual groups is given in parentheses. Thresholds of relative phycocyanin and phycoerythrin fluorescence were used to identify groups of autotrophic cells, and are visualized as black dashed lines. Group A1 maintained mean values of <10, <6, group A2 maintained mean values of >10, <6, group A3 maintained mean values of <10, >6, group A4 maintained mean values of >10, >6 and group A5 maintained mean values of >10, >10 for phycocyanin and phycoerythrin respectively.
(PDF)

S1 Table Physical and chemical parameters in surface samples (top 1m) at stations across transect 1 and transect 2. (PDF)

S2 Table Physical and chemical parameters in depth samples (~30m) at stations across transect 1 and transect 2. (PDF)

S3 Table Correlations between abiotic parameters at (A) ~1m depth and (B) ~30m depth as identified by draftsman plots of pairwise combinations of each parameter (PRIMER). Collinearity threshold was set at r ≥0.90 [50].
(PDF)

S4 Table Total abundance of autotrophic microbial plankton groups A1-A5 and heterotrophic microbial plankton groups LNA and HNA per milliliter of seawater for all stations sampled at the surface (~1m). (PDF)

S5 Table Total abundance of autotrophic microbial plankton groups A1-A5 and heterotrophic microbial plankton groups LNA and HNA per milliliter of seawater for all stations sampled at depth (~30m). (PDF)
