# Supplementary figures and images for: Changes in Microbial Plankton Assemblages Induced by Mesoscale Oceanographic Features in the Northern Gulf of Mexico
Source: PLoS One. 2015 Sep 16;10(9):e0138230. doi: 10.1371/journal.pone.0138230 (PMC4574113; doi:10.1371/journal.pone.0138230)

Figure S1

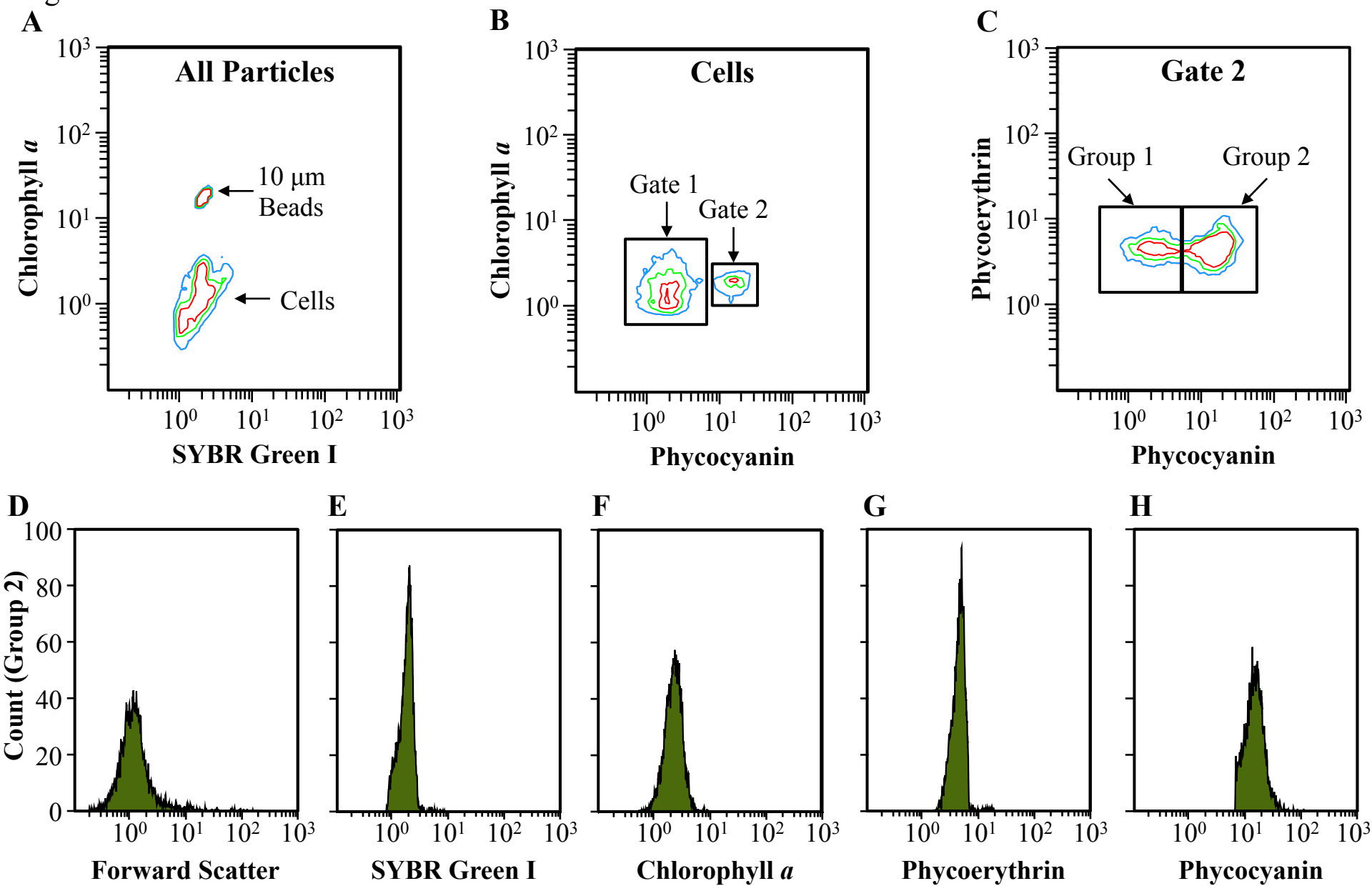

Supplement: S1 Fig — The magnitude of fluorescence is plotted on a logarithmic scale and colored lines represent the percentage of total count: red is 80%, green is 60%, and blue is 40%. (A) All particles counted with cells distinguished from the internal standard of beads. (B) Cells counted are plotted using chlorophyll a and phycocyanin fluorescence. Heterotrophic cells (Gate 1) are distinguished from autotrophic cells (Gate 2). (C) Autotrophic cells are plotted using phycoerytrhin and phycocyanin fluorescence. Groups 1 and 2 are distinguished from each other based on relative phycocyanin fluorescence. (D-H) As an example, we show Group 2 cells plotted against all other measured fluorescence parameters (forward scatter, SYBR Green I, chlorophyll a, phycoerythrin and phycocyanin), to verify consistency in physiological characteristics among individuals. (PDF) [file pone.0138230.s001.pdf]

Figure S2

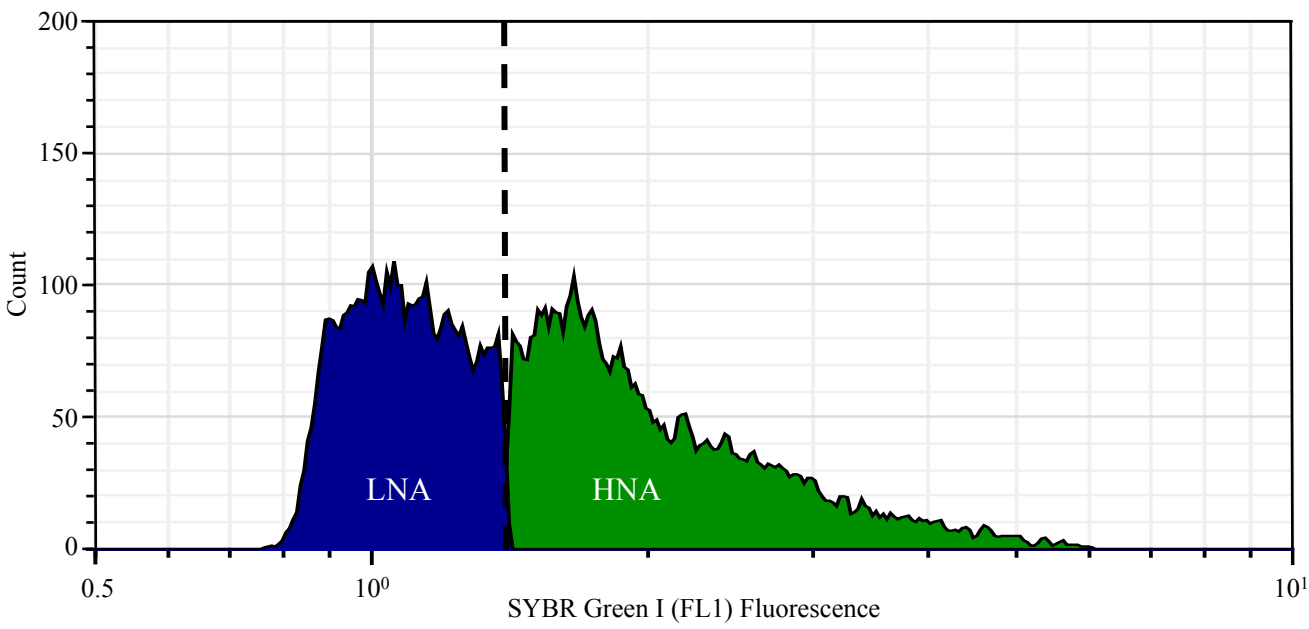

Supplement: S2 Fig — Particle count (y-axis) across the spectrum of SYBR Green I fluorescence (x-axis, plotted in a logarithmic scale) classified as heterotrophic cells using flow cytometry. The delineation between lower-nucleic acid containing bacteria (blue) and relatively higher nucleic acid containing bacteria (green) is marked by a black dashed line. (PDF) [file pone.0138230.s002.pdf]

Fig. S3

**Microbial Signature 1**

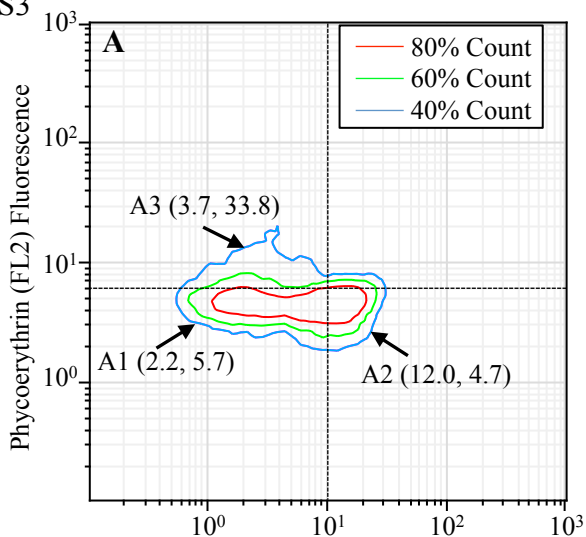

**Microbial Signature 2**

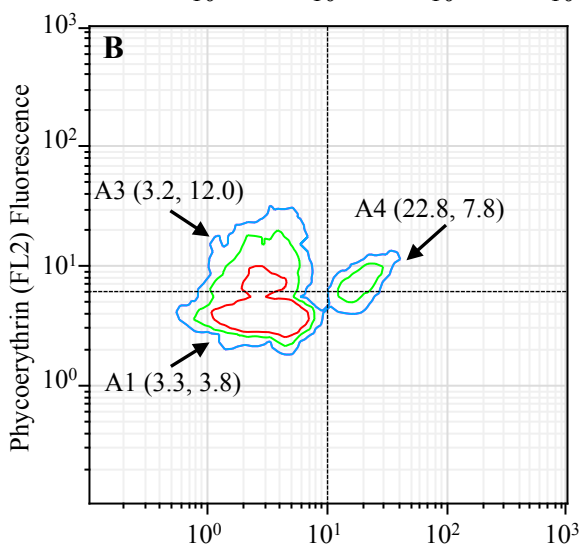

**Microbial Signature 3**

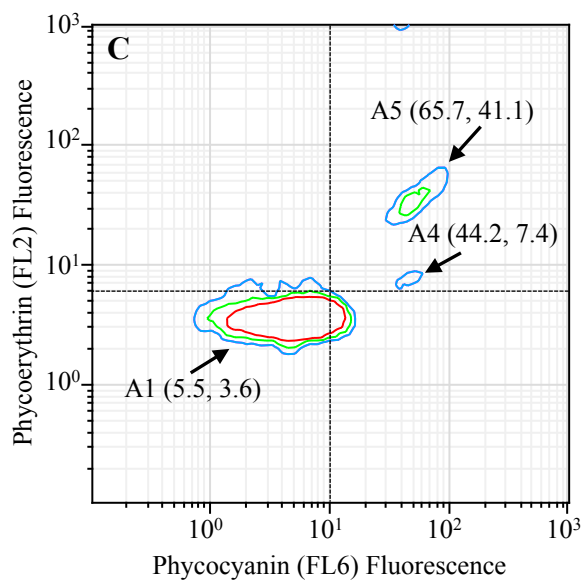

Supplement: S3 Fig — Autotrophic groups in representative samples for three significantly different microbial signatures. The magnitude of fluorescence is plotted on a logarithmic scale (x and y axes) and colored lines represent the percentage of total count. The mean relative phycocyanin fluorescence (x) and mean relative phycoerythrin fluorescence (y) for individual groups is given in parentheses. Thresholds of relative phycocyanin and phycoerythrin fluorescence were used to identify groups of autotrophic cells, and are visualized as black dashed lines. Group A1 maintained mean values of <10, <6, group A2 maintained mean values of >10, <6, group A3 maintained mean values of <10, >6, group A4 maintained mean values of >10, >6 and group A5 maintained mean values of >10, >10 for phycocyanin and phycoerythrin respectively. (PDF) [file pone.0138230.s003.pdf]
