# Supplementary material for: Changes in Microbial Plankton Assemblages Induced by Mesoscale Oceanographic Features in the Northern Gulf of Mexico
Source: PLoS One. 2015 Sep 16;10(9):e0138230. doi: 10.1371/journal.pone.0138230 (PMC4574113; doi:10.1371/journal.pone.0138230)
Supplement: S1 Table — (PDF) [file pone.0138230.s004.pdf]

Station	Temperature °C	Salinity	NO_3^- (µmol L^{-1})	NH_4^+ (µmol L^{-1})	NO_2^- (µmol L^{-1})	Urea (µmol L^{-1})	HPO_4^{2+} (µmol L^{-1})	SiO_2 (µmol L^{-1})	SSH (cm)	Si*	DIN:P
1	29.96	38.51	0.02	0.03	0.01	0.02	0.04	0.03	3.38	0.05	1.31
2	30.60	39.08	0.04	0.00	0.01	0.05	0.10	0.06	2.60	0.05	0.52
3	30.98	39.28	0.06	0.01	0.00	0.01	0.10	0.03	1.97	0.01	0.74
4	30.24	38.93	0.02	0.03	0.01	0.01	0.05	0.03	2.29	0.05	1.08
5	30.08	39.16	0.05	0.03	0.00	0.03	0.10	0.03	4.13	0.02	0.77
6	30.03	39.09	0.03	0.01	0.00	0.01	0.09	0.04	7.81	0.05	0.41
7	30.08	38.69	0.02	0.02	0.00	0.00	0.09	0.04	14.40	0.06	0.43
8	30.46	39.20	0.03	0.01	0.00	0.00	0.08	0.05	23.75	0.06	0.47
9	30.42	38.94	0.04	0.05	0.01	0.02	0.07	0.04	34.20	0.04	1.36
10	30.53	38.80	0.02	0.01	0.00	0.00	0.08	0.06	43.66	0.07	0.38
11	30.14	39.00	0.03	0.01	0.00	0.00	0.09	0.06	49.64	0.07	0.42
12	29.91	38.69	0.02	0.03	0.00	0.01	0.09	0.05	49.42	0.06	0.57
13	29.68	39.54	0.03	0.02	0.00	0.01	0.06	0.04	49.36	0.05	0.76
14	29.72	39.58	0.03	0.03	0.01	0.01	0.09	0.05	20.43	0.06	0.79
15	29.68	39.13	0.04	0.04	0.01	0.04	0.08	0.03	20.84	0.03	1.05
16	29.48	39.10	0.02	0.05	0.01	0.02	0.08	0.03	19.46	0.04	1.13
17	29.59	39.19	0.03	0.04	0.01	0.01	0.09	0.02	16.20	0.03	0.86
18	29.64	39.04	0.03	0.02	0.01	0.00	0.08	0.01	13.04	0.01	0.76
19	28.99	38.05	0.02	0.01	0.00	0.00	0.09	0.05	11.14	0.06	0.42
20	28.19	35.40	0.04	0.03	0.01	0.08	0.12	0.04	10.24	0.03	0.68
21	29.22	36.69	0.03	0.05	0.00	0.04	0.09	0.05	10.33	0.05	0.98
22	29.24	36.14	0.02	0.03	0.00	0.00	0.09	0.03	11.07	0.04	0.62
23	30.33	37.43	0.05	0.08	0.01	0.09	0.13	0.02	11.84	0.00	1.05
24	29.86	37.16	0.02	0.01	0.00	0.01	0.09	0.04	12.19	0.05	0.38
25	30.02	37.59	0.04	0.04	0.00	0.05	0.11	0.08	12.38	0.07	0.82
26	30.16	38.13	0.02	0.01	0.00	0.00	0.10	0.05	12.45	0.07	0.28
