# Supplementary material for: Changes in Microbial Plankton Assemblages Induced by Mesoscale Oceanographic Features in the Northern Gulf of Mexico
Source: PLoS One. 2015 Sep 16;10(9):e0138230. doi: 10.1371/journal.pone.0138230 (PMC4574113; doi:10.1371/journal.pone.0138230)
Supplement: S2 Table — (PDF) [file pone.0138230.s005.pdf]

Station	Salinity	NO_3^- (µmol L^{-1})	NH_4^+ (µmol L^{-1})	NO_2^- (µmol L^{-1})	Urea (µmol L^{-1})	HPO_4^{2+} (µmol L^{-1})	SiO_2 (µmol L^{-1})	SSH (cm)	Si*	DIN:P
1	38.00	0.01	0.02	0.00	0.03	0.05	0.03	3.38	0.04	0.80
2	39.02	0.03	0.03	0.01	0.04	0.05	0.03	2.60	0.03	1.45
3	40.00	0.03	0.02	0.01	0.09	0.22	0.03	1.97	0.03	0.28
4	39.00	0.03	0.02	0.00	0.01	0.09	0.05	2.29	0.04	0.62
5	39.00	0.02	0.03	0.01	0.01	0.06	0.01	4.13	0.02	0.96
6	38.00	0.04	0.05	0.01	0.08	0.06	0.02	7.81	0.01	1.50
7	38.00	0.01	0.02	0.01	0.01	0.06	0.02	14.40	0.04	0.67
8	37.00	0.03	0.03	0.00	0.02	0.07	0.04	23.75	0.03	0.98
9	38.00	0.02	0.02	0.00	0.01	0.06	0.04	34.20	0.04	0.86
10	37.00	0.04	0.02	0.00	0.06	0.18	0.07	43.66	0.05	0.38
11	37.00	0.02	0.02	0.00	0.00	0.08	0.06	49.64	0.06	0.53
12	38.00	0.02	0.01	0.00	0.01	0.07	0.05	49.42	0.05	0.52
13	37.00	0.03	0.01	0.00	0.00	0.08	0.06	49.36	0.05	0.44
14	39.00	0.02	0.02	0.01	0.01	0.08	0.03	20.43	0.04	0.62
15	40.00	0.01	0.01	0.00	0.01	0.08	0.04	20.84	0.06	0.32
16	40.00	0.02	0.02	0.00	0.00	0.09	0.06	19.46	0.06	0.58
17	39.00	0.03	0.10	0.01	0.05	0.08	0.02	16.20	0.02	1.59
18	40.00	0.03	0.04	0.01	0.01	0.07	0.02	13.04	0.01	1.07
19	39.00	0.02	0.03	0.01	0.01	0.09	0.03	11.14	0.04	0.67
20	40.00	0.02	0.02	0.01	0.00	0.09	0.02	10.24	0.03	0.52
21	39.00	0.02	0.01	0.00	0.01	0.10	0.04	10.33	0.05	0.31
22	36.00	0.02	0.03	0.00	0.01	0.09	0.03	11.07	0.04	0.58
23	38.00	0.06	0.06	0.01	0.09	0.14	0.07	11.84	0.04	0.92
24	38.37	0.03	0.03	0.00	0.02	0.10	0.03	12.19	0.03	0.62
25	37.38	0.08	0.13	0.01	0.17	0.15	0.05	12.38	0.00	1.47
26	39.08	0.02	0.01	0.00	0.00	0.11	0.02	12.45	0.03	0.24
