# Supplementary material for: Changes in Microbial Plankton Assemblages Induced by Mesoscale Oceanographic Features in the Northern Gulf of Mexico
Source: PLoS One. 2015 Sep 16;10(9):e0138230. doi: 10.1371/journal.pone.0138230 (PMC4574113; doi:10.1371/journal.pone.0138230)
Supplement: S3 Table — Collinearity threshold was set at r ≥0.90 [50]. (PDF) [file pone.0138230.s006.pdf]

A

	Temp.	Salinity	NO_3^-	HPO_4^{-2}	SiO_2	NH_4^+	NO_2^-	Urea	SSH	Si*	DIN:P
Temp.											
Salinity	0.60										
NO_3^-	0.14	-0.03									
HPO_4^{-2}	-0.12	-0.43	0.53								
SiO_2	0.09	-0.03	-0.17	0.05							
NH_4^+	-0.28	-0.26	0.29	0.11	-0.34						
NO_2^-	-0.34	-0.07	0.21	-0.11	-0.41	0.56					
Urea	-0.22	-0.41	0.65	0.36	-0.07	0.58	0.57				
SSH	-0.02	0.21	-0.24	-0.05	0.34	0.01	-0.29	-0.28			
Si*	-0.12	0.03	-0.70	-0.36	0.76	-0.42	-0.34	-0.43	0.36		
N:P	-0.03	0.08	0.37	-0.35	-0.36	0.72	0.73	0.59	-0.21	-0.40	

B

	Salinity	NO_3^-	HPO_4^{-2}	SiO_2	NH_4^+	NO_2^-	Urea	SSH	Si*	DIN:P
Salinity										
NO_3^-	-0.33									
HPO_4^{-2}	-0.10	0.50								
SiO_2	-0.38	0.41	0.42							
NH_4^+	-0.25	0.64	0.18	-0.09						
NO_2^-	0.17	0.41	0.17	-0.35	0.71					
Urea	-0.21	0.77	0.49	0.14	0.70	0.64				
SSH	-0.43	-0.06	0.00	0.49	-0.19	-0.43	-0.30			
Si*	0.10	-0.58	-0.09	0.42	-0.74	-0.71	-0.65	0.43		
N:P	-0.13	0.45	-0.39	-0.31	0.78	0.59	0.48	-0.24	-0.69	
