# Supplementary material for: Changes in Microbial Plankton Assemblages Induced by Mesoscale Oceanographic Features in the Northern Gulf of Mexico
Source: PLoS One. 2015 Sep 16;10(9):e0138230. doi: 10.1371/journal.pone.0138230 (PMC4574113; doi:10.1371/journal.pone.0138230)
Supplement: S4 Table — (PDF) [file pone.0138230.s007.pdf]

Station	LNA	HNA	A1	A2	A3	A	A5
1	7.02×10^4	1.47×10^5	5.00×10^4	BDL	2.08×10^4	2.63×10^3	1.15×10^4
2	1.48×10^5	1.58×10^5	5.31×10^4	BDL	1.69×10^4	2.45×10^3	1.24×10^4
3	1.38×10^5	1.03×10^5	5.98×10^4	BDL	3.27×10^4	8.44×10^2	1.37×10^4
4	1.12×10^5	9.30×10^4	4.84×10^4	BDL	1.96×10^4	1.41×10^3	1.03×10^4
5	1.07×10^5	1.57×10^5	6.31×10^4	BDL	4.02×10^4	9.40×10^3	1.61×10^4
6	1.67×10^5	1.35×10^5	5.45×10^4	BDL	2.64×10^4	6.14×10^3	1.43×10^4
7	8.73×10^4	1.60×10^5	3.57×10^4	BDL	2.91×10^4	1.73×10^3	1.09×10^4
8	6.31×10^4	1.63×10^5	4.05×10^4	BDL	1.41×10^4	1.91×10^3	1.50×10^4
9	5.23×10^4	1.60×10^5	1.20×10^5	BDL	1.19×10^4	7.23×10^2	6.99×10^3
10	8.02×10^4	7.45×10^4	4.96×10^4	BDL	1.67×10^4	1.92×10^2	4.71×10^3
11	5.28×10^4	1.03×10^5	4.32×10^4	BDL	1.46×10^4	3.34×10^2	4.26×10^3
12	4.38×10^4	1.09×10^5	5.12×10^4	BDL	7.98×10^3	7.69×10^2	4.32×10^3
13	5.01×10^4	1.13×10^5	6.40×10^4	BDL	4.27×10^3	5.17×10^2	4.76×10^3
14	5.48×10^4	1.31×10^5	8.33×10^4	BDL	1.09×10^4	1.26×10^3	4.49×10^3
15	1.03×10^5	1.36×10^5	7.17×10^4	BDL	1.89×10^4	2.37×10^3	1.38×10^4
16	6.36×10^4	1.57×10^5	6.84×10^4	BDL	1.31×10^4	2.42×10^3	9.73×10^3
17	6.63×10^4	1.14×10^5	8.93×10^4	BDL	7.71×10^3	2.45×10^3	6.25×10^3
18	6.65×10^4	1.14×10^5	1.00×10^5	BDL	1.71×10^4	3.33×10^3	6.02×10^3
19	3.48×10^5	1.63×10^5	3.12×10^4	9.43×10^4	4.15×10^3	2.17×10^4	1.13×10^4
20	3.58×10^5	2.46×10^5	4.60×10^4	9.00×10^3	3.08×10^3	3.68×10^4	1.08×10^4
21	3.11×10^4	1.12×10^5	2.71×10^4	5.25×10^4	4.06×10^3	1.65×10^3	3.73×10^3
22	6.27×10^4	1.92×10^5	6.96×10^4	9.61×10^4	5.02×10^3	9.85×10^3	7.56×10^3
23	2.23×10^5	2.28×10^5	1.07×10^5	1.02×10^5	7.12×10^3	1.59×10^4	7.26×10^3
24	9.70×10^4	2.36×10^5	6.62×10^4	8.66×10^4	3.12×10^3	4.33×10^3	5.34×10^3
25	7.25×10^4	1.92×10^5	4.66×10^4	1.01×10^5	3.29×10^3	1.10×10^4	7.31×10^3
26	7.05×10^4	1.46×10^5	7.16×10^4	5.48×10^4	3.01×10^3	1.48×10^3	3.06×10^3
