# Supplementary material for: Changes in Microbial Plankton Assemblages Induced by Mesoscale Oceanographic Features in the Northern Gulf of Mexico
Source: PLoS One. 2015 Sep 16;10(9):e0138230. doi: 10.1371/journal.pone.0138230 (PMC4574113; doi:10.1371/journal.pone.0138230)
Supplement: S5 Table — (PDF) [file pone.0138230.s008.pdf]

Station	LNA	HNA	A1	A2	A3	A4	A5
1	4.38×10^4	9.23×10^4	9.16×10^4	BDL	8.12×10^3	1.51×10^3	5.89×10^2
2	1.16×10^5	7.57×10^4	9.10×10^4	BDL	9.61×10^3	3.05×10^2	8.21×10^2
3	4.71×10^4	1.16×10^5	1.18×10^5	BDL	2.80×10^4	1.01×10^3	2.47×10^3
4	6.69×10^4	9.66×10^4	8.80×10^4	BDL	1.02×10^4	8.03×10^2	5.80×10^2
5	8.05×10^4	1.20×10^5	1.39×10^5	BDL	7.83×10^3	1.09×10^3	4.30×10^3
6	2.17×10^5	9.12×10^4	6.00×10^4	BDL	1.30×10^4	4.30×10^3	6.34×10^2
7	8.78×10^4	1.80×10^5	3.19×10^4	BDL	1.55×10^3	1.05×10^4	5.03×10^3
8	2.47×10^4	1.49×10^5	3.60×10^4	BDL	3.81×10^3	1.24×10^4	5.05×10^3
9	2.56×10^4	2.28×10^5	1.51×10^5	BDL	1.21×10^4	4.86×10^3	2.16×10^3
10	5.15×10^4	1.85×10^5	5.11×10^4	BDL	4.69×10^3	1.85×10^3	4.56×10^2
11	2.17×10^4	1.47×10^5	4.29×10^4	BDL	4.98×10^3	1.14×10^3	2.71×10^3
12	1.98×10^4	1.40×10^5	5.98×10^4	BDL	8.46×10^3	1.93×10^3	1.74×10^3
13	5.81×10^4	1.28×10^5	8.40×10^4	BDL	5.35×10^3	1.05×10^4	7.64×10^2
14	3.87×10^4	7.52×10^4	6.32×10^4	BDL	9.34×10^3	1.40×10^2	2.94×10^3
15	9.39×10^4	1.30×10^5	4.94×10^4	BDL	7.83×10^3	1.25×10^3	1.37×10^4
16	4.03×10^4	1.28×10^5	7.31×10^4	BDL	7.66×10^3	5.50×10^2	5.68×10^3
17	6.41×10^4	1.11×10^5	9.42×10^4	BDL	5.78×10^3	2.74×10^3	9.63×10^3
18	2.78×10^5	4.02×10^5	1.32×10^5	BDL	3.63×10^4	5.47×10^3	9.60×10^3
19	1.21×10^5	1.03×10^5	6.82×10^4	BDL	4.34×10^3	1.57×10^3	8.15×10^3
20	5.56×10^4	1.22×10^5	7.23×10^4	BDL	6.36×10^3	8.88×10^2	6.94×10^3
21	2.17×10^4	8.70×10^4	7.08×10^4	BDL	6.35×10^3	1.04×10^3	4.75×10^3
22	2.00×10^4	1.42×10^5	6.58×10^4	BDL	6.09×10^3	1.28×10^3	1.18×10^4
23	7.79×10^4	1.42×10^5	4.74×10^4	BDL	7.44×10^3	3.47×10^3	1.17×10^4
24	6.90×10^4	1.84×10^5	1.53×10^5	BDL	7.92×10^3	4.64×10^3	8.98×10^3
25	4.94×10^4	1.92×10^5	1.28×10^5	BDL	8.22×10^3	6.12×10^3	3.53×10^3
26	ND	ND	ND	ND	ND	ND	ND
